# Supplementary material for: User Perceptions of Visual Blood: An International Mixed Methods Study on Novel Blood Gas Analysis Visualization
Source: Diagnostics (Basel). 2023 Sep 30;13(19):3103. doi: 10.3390/diagnostics13193103 (PMC10572252; doi:10.3390/diagnostics13193103)
Supplement: Supplementary file 1 [file diagnostics-13-03103-s001.zip › File S1.pdf]

Participant 1

POSITIVE: What did you <u>LIKE</u> about Visual Blood?

1.1 [Easy to use]
1.2 [Fast to learn]
1.3 [Clear presentation of complex disorders]
1.4 [Malfunctions can be detected more effectively and faster]

NEGATIVE: What did you *DISLIKE* about Visual Blood?

1.5 [it is difficult to assign oxygen parameters]

Participant 2

POSITIVE: What did you <u>LIKE</u> about Visual Blood?

2.1 [Faster overview of respiratory/metabolic overall situation than with conventional blood gas analysis]
2.2 [fewer values are forgotten to look at]

NEGATIVE: What did you *DISLIKE* about Visual Blood?

2.3 [Nevertheless, the interpretation time of 15s is a bit short]
2.4 [absolute values necessary in addition]

Participant 3

POSITIVE: What did you <u>LIKE</u> about Visual Blood?

3.1 [overview]

NEGATIVE: What did you *DISLIKE* about Visual Blood?

3.2 [Distraction like the Peace Flag]

Participant 4

POSITIVE: What did you LIKE about Visual Blood?

[(Nothing entered)]

NEGATIVE: What did you *DISLIKE* about Visual Blood?

4.1 [Confusing that here the flashing/dashed line means something different than in the Visual Patient.]
4.2 [Found the second view screen with the enlarged scale confusing.]

Participant 5

POSITIVE: What did you LIKE about Visual Blood?

(Nothing entered)

NEGATIVE: What did you *DISLIKE* about Visual Blood?

(Nothing entered)

Participant 6

POSITIVE: What did you LIKE about Visual Blood?

(Nothing entered)

NEGATIVE: What did you *DISLIKE* about Visual Blood?

(Nothing entered)

Participant 7

POSITIVE: What did you <u>LIKE</u> about Visual Blood?

7.1 [Obviousness «hypo «versus «hyper» animation]

NEGATIVE: What did you *DISLIKE* about Visual Blood?

7.2 [Design needs improvement]
7.3 [Flashing partially too discreet]

Participant 8

POSITIVE: What did you LIKE about Visual Blood?

8.1 [Normal blood gas analysis can be identified quickly]

NEGATIVE: What did you *DISLIKE* about Visual Blood?

8.2 [The more complex the blood gas results are, the better I think the conventional blood gas analysis is]

Participant 9

POSITIVE: What did you LIKE about Visual Blood?

(Nothing entered)

NEGATIVE: What did you *DISLIKE* about Visual Blood?

(Nothing entered)

Participant 10

POSITIVE: What did you LIKE about Visual Blood?

10.1 [more illustrative than a conventional blood gas analysis]

NEGATIVE: What did you *DISLIKE* about Visual Blood?

10.2 [but significantly less accurate]
10.3 [if there are too many values outside the normal range, the hullabaloo becomes a bit too much]
10.4 [the flashing over there, distracts from the flashing over here]

Participant 11

POSITIVE: What did you LIKE about Visual Blood?

11.1 [Lactate visualization is very good and fast recognizable]

NEGATIVE: What did you *DISLIKE* about Visual Blood?

11.2 [Oxygen representation]
11.3 [Hemoglobin representation]

Participant 12

POSITIVE: What did you LIKE about Visual Blood?

12.1 [Very interesting]
12.2 [Faster detection]

NEGATIVE: What did you *DISLIKE* about Visual Blood?

12.3 [still needs optimization]

Participant 13

POSITIVE: What did you LIKE about Visual Blood?

13.1 [Visually appealing]
13.2 [colorful]
13.3 [Principle of "too much/less" quickly understood]

NEGATIVE: What did you *DISLIKE* about Visual Blood?

13.4 [A bit too much sometimes]

Participant 14

POSITIVE: What did you LIKE about Visual Blood?

14.1 [cool]
14.2 [innovative]
14.3 [fun to use]

NEGATIVE: What did you *DISLIKE* about Visual Blood?

14.4 [Dichotomy]
14.5 [Pre-interpretation, how are the limits defined]
14.6 [too inaccurate for the thorax operating room for example]
14.7 [Blood gas analysis with red markings would be better]

Participant 15

POSITIVE: What did you LIKE about Visual Blood?

15.1 [After thinking about it for a moment, logically]
15.2 [Fun to use]
15.3 [Lactate etc "funny, illustrative" presented]
15.4 [Peace flag is cool]

NEGATIVE: What did you *DISLIKE* about Visual Blood?

15.5 [Visualization of erythrocytes can be further improved]

Participant 16

POSITIVE: What did you LIKE about Visual Blood?

16.1 [Faster overview of what is going on]
16.2 [Time saving]
16.3 [visually appealing]

NEGATIVE: What did you *DISLIKE* about Visual Blood?

16.4 [Quantity is missing]

Participant 17

POSITIVE: What did you LIKE about Visual Blood?

17.1 [Gamification]
17.2 [unified presentation]

NEGATIVE: What did you *DISLIKE* about Visual Blood?

17.3 [Slow speed of the individual components swimming through]

Participant 18

POSITIVE: What did you LIKE about Visual Blood?

18.1 [Quick detection of the main problem]

NEGATIVE: What did you *DISLIKE* about Visual Blood?

18.2 [In mixed disorders, not clear which alteration is more important]

Participant 19

POSITIVE: What did you LIKE about Visual Blood?

19.1 [Animation]
19.2 [overview at a first glance]
19.3 [fun to watch]

NEGATIVE: What did you *DISLIKE* about Visual Blood?

19.4 [No absolute values]
19.5 [must still look at a conventional blood gas analysis afterwards]
19.6 [very much information on a small space]

Participant 20

POSITIVE: What did you LIKE about Visual Blood?

20.1 [Faster intuitive analysis possible]

NEGATIVE: What did you *DISLIKE* about Visual Blood?

20.2 [No quantitative assessment]
20.3 [Too much motion]

Participant 21

POSITIVE: What did you LIKE about Visual Blood?

21.1 [It is fun]

NEGATIVE: What did you *DISLIKE* about Visual Blood?

21.2 [You have to look quite long to get an overview, especially if several parameters are together]

Participant 22

POSITIVE: What did you LIKE about Visual Blood?

22.1 [Excitingly designed]
22.2 [Good visualization]
22.3 [Electrolytes simple]
22.4 [great as a supplement (split screen)]

NEGATIVE: What did you *DISLIKE* about Visual Blood?

22.5 [Needs to get used to it]

Participant 23

POSITIVE: What did you LIKE about Visual Blood?

23.1 [You get to the main diagnosis quickly]

NEGATIVE: What did you *DISLIKE* about Visual Blood?

23.2 [Numbers are missing]
23.3 [would be even more useful if it says the diagnosis directly]

Participant 24

POSITIVE: What did you LIKE about Visual Blood?

24.1 [concept]

NEGATIVE: What did you *DISLIKE* about Visual Blood?

24.2 [Graphic]

Participant 25

POSITIVE: What did you LIKE about Visual Blood?

(nothing entered)

NEGATIVE: What did you *DISLIKE* about Visual Blood?

25.1 [too much in one picture]
25.2 [quantitative is missing]

Participant 26

POSITIVE: What did you LIKE about Visual Blood?

26.1 [Quick distinction between normal/abnormal values]

NEGATIVE: What did you *DISLIKE* about Visual Blood?

26.2 [Not quantitative]

Participant 27

POSITIVE: What did you LIKE about Visual Blood?

27.1 [innovative representation of complex information]

NEGATIVE: What did you *DISLIKE* about Visual Blood?

(nothing entered)

Participant 28

POSITIVE: What did you LIKE about Visual Blood?

28.1 [You can see much faster when a parameter is out of the normal range]
28.2 [faster than on paper]

NEGATIVE: What did you *DISLIKE* about Visual Blood?

28.3 [More difficult to learn than Visual Patient (less intuitive, more challenging)]
28.4 [appears turbulent (everything is moving)]
28.5 [On the PC, the normal blood gas analyses are colored (red values outside the norm, cave bias).]

Participant 29

POSITIVE: What did you LIKE about Visual Blood?

29.1 [It is fun.]

NEGATIVE: What did you *DISLIKE* about Visual Blood?

29.2 [There are a lot of components at the same time and therefore it is sometimes difficult to detect abnormal components.]
29.3 [Some colors are the same, e.g. desaturated hemoglobin and potassium. Which makes it sometimes confusing.]

Participant 30

POSITIVE: What did you LIKE about Visual Blood?

30.1 [Basic concept]

NEGATIVE: What did you *DISLIKE* about Visual Blood?

30.2 [Design: floating through information, redundant information. I prefer stationary and separate.]
30.3 [Less flashing and bubbling.]
30.4 [Arrange groupings/connections more clearly, e.g. gases together.]
30.5 [Possibly size differences for pathologies.]

Participant 31

POSITIVE: What did you LIKE about Visual Blood?

31.1 [witty]
31.2 [quite easy to understand]

NEGATIVE: What did you *DISLIKE* about Visual Blood?

31.3 [Velocity]
31.4 [more appealing graphics]

Participant 32

POSITIVE: What did you LIKE about Visual Blood?

32.1 [Great add-on to the conventional display method.]
32.2 [Helps to see things more easily if you are a visual type.]

NEGATIVE: What did you *DISLIKE* about Visual Blood?

32.3 [Proportions (some things too big, e.g. scale takes a lot of space even if in physiologically state)]
32.4 [Velocity of the "blood flow" too slow.]
32.5 [Still looks a bit trashy ;)]

Participant 33

POSITIVE: What did you LIKE about Visual Blood?

33.1 [Good overview]
33.2 [Everything at a glance]

NEGATIVE: What did you *DISLIKE* about Visual Blood?

33.3 [A bit hectic]

Participant 34

POSITIVE: What did you LIKE about Visual Blood?

34.1 [Beautiful 3D visualization.]
34.2 [It quickly becomes apparent which parameters are out of the normal range.]

NEGATIVE: What did you *DISLIKE* about Visual Blood?

34.3 [Flow of molecules was too fast.]
34.4 [It is too dynamic.]

Participant 35

POSITIVE: What did you LIKE about Visual Blood?

(nothing entered)

NEGATIVE: What did you *DISLIKE* about Visual Blood?

35.1 [Overall picture is turbulent]

Participant 36

POSITIVE: What did you LIKE about Visual Blood?

36.1 [intuitive]
36.2 [Logical and consistent visualization]
36.3 [good learning curve at the beginning]

NEGATIVE: What did you *DISLIKE* about Visual Blood?

36.4 [Rotation e.g. with the electrolyte, makes it sometimes more difficult to observe]
36.5 [pH value representation could be more clear]

Participant 37

POSITIVE: What did you LIKE about Visual Blood?

37.1 [inventive way of presentation]
37.2 [mostly detectable at first glance]

NEGATIVE: What did you *DISLIKE* about Visual Blood?

37.3 [Some representations still need to be interpreted and are not intuitive.]

Participant 38

POSITIVE: What did you LIKE about Visual Blood?

38.1 [Lactate milk bottle]

NEGATIVE: What did you *DISLIKE* about Visual Blood?

38.2 [High level of simultaneous stimuli in different areas of the field of view]

Participant 39

POSITIVE: What did you LIKE about Visual Blood?

(Nothing entered)

NEGATIVE: What did you *DISLIKE* about Visual Blood?

(Nothing entered)

Participant 40

POSITIVE: What did you <u>LIKE</u> about Visual Blood?

40.1 [Illustration]

NEGATIVE: What did you *DISLIKE* about Visual Blood?

40.2 [If too many parameters are out of the normal range it gets confusing]

Participant 41

POSITIVE: What did you <u>LIKE</u> about Visual Blood?

41.1 [moderate]

NEGATIVE: What did you *DISLIKE* about Visual Blood?

41.2 [Rapid flashing has a stressful effect.]
41.3 [Combined disorders look confusing.]
41.4 [For CO2 I would have liked to see molecule models known from school]

Participant 42

POSITIVE: What did you LIKE about Visual Blood?

42.1 [creative colorful varied presentation]
42.2 [Fast overview over pathological results]

NEGATIVE: What did you *DISLIKE* about Visual Blood?

42.3 [No absolute values]
42.4 [Pathological values that are too low are " missed" at first sight]

Participant 43

POSITIVE: What did you <u>LIKE</u> about Visual Blood?

(nothing entered)

43.1 [I can well imagine that one can understand the BGA better with it then.]

NEGATIVE: What did you *DISLIKE* about Visual Blood?

43.2 [The introduction was too short for me. However, I didn't notice this until the test videos started.]

43.3 [It would be nice to have a support menu.]

Participant 44

POSITIVE: What did you LIKE about Visual Blood?

44.1 [Everything at a glance]

NEGATIVE: What did you *DISLIKE* about Visual Blood?

44.2 [Quantification not provided]

Participant 45

POSITIVE: What did you LIKE about Visual Blood?

45.1 [Playful evaluation of the blood gas analysis]
45.2 [Visualization]

NEGATIVE: What did you *DISLIKE* about Visual Blood?

45.3 [Difficult to remember everything during the introduction video]

Participant 46

POSITIVE: What did you LIKE about Visual Blood?

46.1 [Good memorizing ability thanks to visualization]

NEGATIVE: What did you *DISLIKE* about Visual Blood?

46.2 [Too many pictures, very disorganized]

Participant 47

POSITIVE: What did you LIKE about Visual Blood?

47.1 [Simultaneous seeing and perception of pathologically altered blood gas analyses]

NEGATIVE: What did you *DISLIKE* about Visual Blood?

47.2 [Definitions of pathological versus normal states after watching the introduction video only once not clearly remembered for all parameters]

Participant 48

POSITIVE: What did you LIKE about Visual Blood?

48.1 [Good overview over all values]
48.2 [Covered several values at once with just a few symbols.]
48.3 [Pathologies in particular highlighted.]

NEGATIVE: What did you *DISLIKE* about Visual Blood?

48.4 [Due to movement sometimes symbols are not in the screen for a few seconds, this might cause information to get lost.]

Participant 49

POSITIVE: What did you LIKE about Visual Blood?

49.1 [Unified visual representation for too high/low]

NEGATIVE: What did you *DISLIKE* about Visual Blood?

49.2 [Display of pH value, oxygen saturation and oxygen affinity too inconspicuous]

Participant 50

POSITIVE: What did you <u>LIKE</u> about Visual Blood?

50.1 [Variety]
50.2 [Blood gas analysis without numbers]

NEGATIVE: What did you *DISLIKE* about Visual Blood?

50.3 [With many pathological parameters, an overview is more difficult in 15 seconds]

Participant 51

POSITIVE: What did you LIKE about Visual Blood?

51.1 [Flashing parameters]

NEGATIVE: What did you *DISLIKE* about Visual Blood?

51.2 [Too much input when many values are altered]

Participant 52

POSITIVE: What did you LIKE about Visual Blood?

52.1 [Faster detection of pathological values]

NEGATIVE: What did you *DISLIKE* about Visual Blood?

52.2 [No structured going through in sequence, possibly overlooking of pathologically altered values, because other, also pathological values are more dominant]

Participant 53

POSITIVE: What did you LIKE about Visual Blood?

(nothing entered)

NEGATIVE: What did you *DISLIKE* about Visual Blood?

(nothing entered)

Participant 54

POSITIVE: What did you LIKE about Visual Blood?

54.1 [Complex representation that can be easily interpreted]

NEGATIVE: What did you *DISLIKE* about Visual Blood?

54.2 [Not knowing what to expect, I was inattentive at the beginning of the educational video. Important content was lost as a result.]

Participant 55

POSITIVE: What did you LIKE about Visual Blood?

55.1 [Structure]

NEGATIVE: What did you *DISLIKE* about Visual Blood?

55.2 [That one could not always see all values at the same time and thus had to "wait" for some values.]

Participant 56

POSITIVE: What did you LIKE about Visual Blood?

56.1 [Fun]
56.2 [something new]

NEGATIVE: What did you *DISLIKE* about Visual Blood?

56.3 [When more than two things are pathological, it gets difficult to capture everything.]

Participant 57

POSITIVE: What did you LIKE about Visual Blood?

57.1 [Visualization]
57.2 [new approach to quickly evaluate a blood gas analysis]

NEGATIVE: What did you *DISLIKE* about Visual Blood?

57.3 [Display of acid-base balance could be better]

Participant 58

POSITIVE: What did you LIKE about Visual Blood?

58.1 [The scale is cool]
58.2 [Significance becomes clear quickly]
58.3 [Flashing you get immediately]
58.4 [Icons are very good (e.g. milk bottle and carbon monoxide)]

NEGATIVE: What did you *DISLIKE* about Visual Blood?

58.5 [Not always everything visible]
58.6 [Erythrocyte presentation not always easy to recognize]

Participant 59

POSITIVE: What did you LIKE about Visual Blood?

59.1 [You notice more quickly what is conspicuous]
59.2 [easy to learn]
59.3 [Blood gas analysis is easier to imagine than Visual Clot]

NEGATIVE: What did you *DISLIKE* about Visual Blood?

59.4 [Time pressure]
59.5 [a legend for Visual Blood would be helpful]

Participant 60

POSITIVE: What did you <u>LIKE</u> about Visual Blood?

60.1 [Clear code for individual parameters and their changes]

NEGATIVE: What did you *DISLIKE* about Visual Blood?

60.2 [Inconvenient, confusing.]
60.3 [Pathological changes can be missed]
60.4 [No quantitative information about the changes.]

Participant 61

POSITIVE: What did you LIKE about Visual Blood?

(nothing entered)

NEGATIVE: What did you *DISLIKE* about Visual Blood?

(nothing entered)

Participant 62

POSITIVE: What did you <u>LIKE</u> about Visual Blood?

(nothing entered)

NEGATIVE: What did you *DISLIKE* about Visual Blood?

(nothing entered)

Participant 63

POSITIVE: What did you LIKE about Visual Blood?

63.1 [Good graphical representation]

NEGATIVE: What did you *DISLIKE* about Visual Blood?

63.2 [The passing by of the individual parameters -> possibility to fix them in the picture?]

Participant 64

POSITIVE: What did you LIKE about Visual Blood?

(Nothing entered)

NEGATIVE: What did you *DISLIKE* about Visual Blood?

64.1 [Sometimes with complex disorders you have to pay attention to many things and the velocity for that is a bit too high]

Participant 65

POSITIVE: What did you LIKE about Visual Blood?

65.1 [With a bit of practice, pathologies can probably be detected more quickly/ at first glance than with conventional blood gas analysis]

NEGATIVE: What did you *DISLIKE* about Visual Blood?

65.2 [Too overloaded and confusing when many parameters are out of the normal range]

Participant 66

POSITIVE: What did you LIKE about Visual Blood?

66.1 [Graphic is appealing]
66.2 [Values that are rarely observed receive more attention]

NEGATIVE: What did you *DISLIKE* about Visual Blood?

66.3 [Confusing, as all values are displayed at one time]
66.4 [which requires practice]
66.5 [Hypoxia is difficult to detect]

Participant 67

POSITIVE: What did you LIKE about Visual Blood?

67.1 [Fast overview]

NEGATIVE: What did you *DISLIKE* about Visual Blood?

67.2 [Missing out individual parameters]

Participant 68

POSITIVE: What did you LIKE about Visual Blood?

68.1 [Graphic representation: too much and too little were well represented]

NEGATIVE: What did you *DISLIKE* about Visual Blood?

68.2 [Precision of the presentation anemia, oxygen saturation, oxygen affinity, oxygen semi-saturation.]

Participant 69

POSITIVE: What did you LIKE about Visual Blood?

69.1 [States of less and too much can be identified well]
69.2 [The scale explains the pH value well]
69.3 [Base excess, etc., osmolarity is good to grab]

NEGATIVE: What did you *DISLIKE* about Visual Blood?

69.4 [Very crowded, agitated]
69.5 [Condition hypernatremia not recognized]

Participant 70

POSITIVE: What did you <u>LIKE</u> about Visual Blood?

70.1 [it is manageable/straighforward)]
70.2 [it is quite easy to use]

NEGATIVE: What did you *DISLIKE* about Visual Blood?

(Nothing entered)
